# Intrathecal treatment trial of rituximab in progressive MS: results after a 2-year extension

**DOI:** 10.1007/s00415-020-10210-0

**Published:** 2020-09-08

**Authors:** Joakim Bergman, Joachim Burman, Tommy Bergenheim, Anders Svenningsson

**Affiliations:** 1 Department of Clinical Science, Umeå University, Umeå, Sweden; 2 Department of Neurosciences, Uppsala University, Uppsala, Sweden; 3 Department of Clinical Sciences, Karolinska Institutet Danderyd Hospital, Danderyd Hospital, 182 88 Stockholm, Sweden

**Keywords:** Multiple sclerosis, Rituximab, Intrathecal, Progressive MS, Treatment, Clinical trial

## Abstract

**Objectives:**

To evaluate the effect of intrathecally (IT) delivered rituximab as a therapeutic intervention for progressive multiple sclerosis (PMS) during a 3-year follow-up period.

**Methods:**

Participants of a 1-year open-label phase 1b study of IT delivered rituximab to patients with PMS were offered extended treatment with follow-up for an additional 2 years. During the extension phase, treatment with 25 mg rituximab was administered every 6 months via a subcutaneous Ommaya reservoir connected to the right frontal horn with a ventricular catheter.

**Results:**

Mild to moderate vertigo and nausea occurred in 4 out of 14 participants as temporary adverse events associated with IT rituximab infusion. During the entire 3-year period, two cases of low-virulent bacterial meningitis occurred, which were successfully treated. Walking speed deteriorated significantly during the study.

**Conclusions:**

IT administration of rituximab via a ventricular catheter was well tolerated. Considering the meningitis cases, the risk of infection was not negligible. The continued loss of walking speed indicates that IT rituximab was not able to stop disease progression.

**Classification of evidence:**

This study provides class IV evidence that intraventricularly administered rituximab in progressive MS is associated with a risk for bacterial meningitis and does not halt disease progression.

**EU Clinical Trial Register:**

EudraCT; 2008-002626-11 and 2012-000721-53

## Introduction

B-cells located within the CNS constitute a potential target for treatment in progressive MS (PMS) [7]. Administration of B cell depleting antibodies are highly efficacious in the relapsing phase but results in progressive MS (PMS) have been less convincing [5, 6, 8]. Low penetrance to the CNS compartment may explain the limited treatment effect in PMS [6, 8]. Therapeutic antibodies used in the treatment of RRMS does not readily cross the intact blood–brain barrier (BBB), achieving cerebrospinal fluid (CSF) concentrations of only 0.1–0.5% of the corresponding levels in plasma [9]. Intrathecal (IT) administration of rituximab might, therefore, be beneficial for PMS. We conducted a 1-year phase 1b Intrathecal Treatment Trial in Progressive Multiple Sclerosis (ITT-PMS) study on a group of PMS patients either failing, or not benefitting from, existing immunomodulatory treatments confirming the feasibility and tolerability of that treatment approach [1]. The short-term follow-up of that trial precluded the detection of any disease-modifying effect on disease progression. After an additional 2-year extension study, we have here evaluated the potential clinical benefit of rituximab over the total 3-year period.

## Materials and methods

### Ethical approval of standard protocols, registrations, and patient consent

The original ITT-PMS study and the extension study were both approved by the Regional Ethical Review Board in Umeå and registered with the EU Clinical Trial Register (EudraCT; 2008-002626-11 and 2012-000721-53, respectively). The trials were performed in accordance with standards of GCP and the principles of the Declaration of Helsinki. Oral and written information about the trials were provided to all participants before written consent was obtained. The studies were monitored by an independent medical monitor.

### Study cohort

**Fig. 1:**
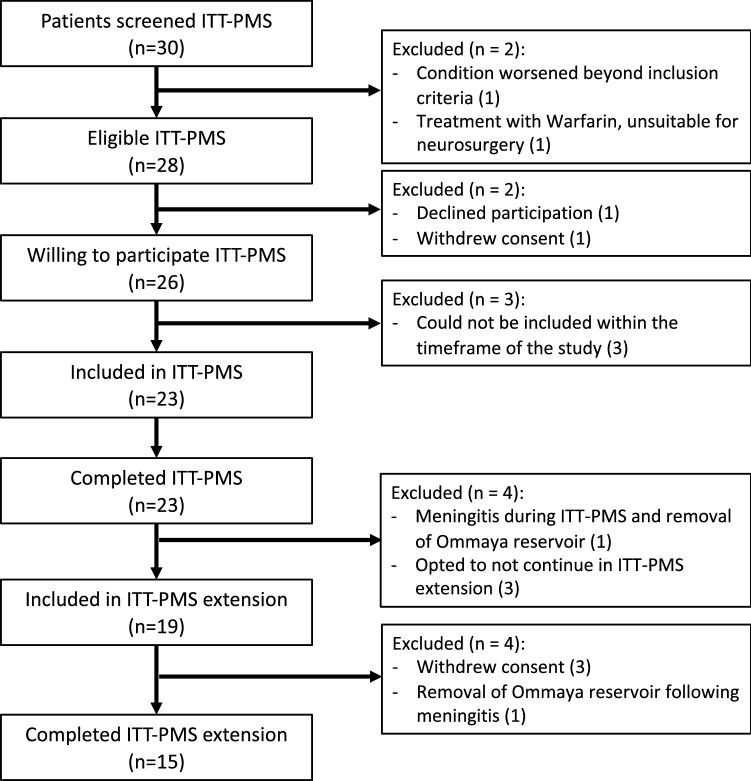
Flow chart of inclusion and exclusion of patients for the combined trials ITT-PMS and ITT-PMS extension

The recruitment to ITT-PMS and ITT-PMS extension trials is depicted in Fig. 1. Eligible patients had a purely progressive MS, where no available treatment options were considered to be beneficial for the patient. Participants were recruited at the neurology departments of Norrland’s University Hospital, Umeå and Uppsala Hospital, Sweden between 27th June 2009 and 11th May 2015. Inclusion- and exclusion criteria are described in detail elsewhere [1]. Baseline characteristics for both studies are shown in Table 1.

**Table 1:** Demographics and disease characteristics

	ITT-PMS (*n* = 23)	ITT-PMS extension (*n* = 15)
Age at inclusion, years		
Mean (SD)	46 (9)	47 (9)
Min–Max	29–66	29–66
Sex, *n* (%)		
Male	7 (30)	4 (27)
Female	16 (70)	11 (73)
Age at disease onset, years		
Mean (SD)	32 (11)	32 (12)
Min–Max	12–51	12–51
Disease duration at inclusion, years		
Mean (SD)	14 (8)	15 (9)
Min–Max	3–39	3–39
Age at PMS onset, years		
Mean (SD)	38 (9)	40 (9)
Min–Max	25–56	25–56
Duration with PMS at inclusion, years		
Mean (SD)	8 (4)	8 (4)
Min–Max	3–19	3–19
Type of PMS, *n* (%)		
SPMS	15 (65)	11 (73)
PPMS	8 (35)	4 (27)
EDSS at inclusion		
Median (IQR)	6.5 (1.0)	6.5 (1.0)
Min–Max	4.0–7.5	4.0–7.0

Demographic features and disease characteristics at baseline in the ITT-PMS trial for the 23 participants in the ITT-PMS trial and the 15 participants that completed the 2-year extension study

### Study design and outcome measures

After completion of the ITT-PMS study, participants were offered to continue treatment in the open-label unblinded extension study. The primary endpoints were to evaluate stabilization of neurological deterioration, degree of MS symptoms, quality of life, and fatigue.

### Study procedures

#### Intrathecal (IT) access

In the original ITT-PMS study, participants received an Ommaya reservoir as an access point for intraventricular injections [1].

#### Treatment within the study

**Fig. 2:**
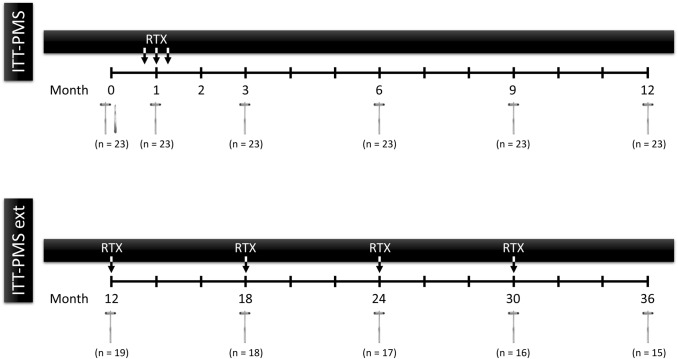
Overview of the study design of the ITT-PMS and the ITT-PMS extension trials. Treatment within the trials are marked with ‘RTX’. Month 12 was the last visit in the ITT-PMS trial and at the same time the first visit in the ITT-PMS extension trial with treatment according to the protocols of the extension trial. The reflex hammers indicate the timing of clinical evaluation. The scalpel indicates the insertion of the Ommaya reservoir. *ITT-PMS* Intrathecal Treatment Trial in Progressive Multiple Sclerosis, *ITT-PMS ext* Intrathecal Treatment Trial in Progressive Multiple Sclerosis extension, *RTX* rituximab

Initial treatment in the ITT-PMS trial was three injections of 25 mg rituximab (Mabthera^®^) 1 week apart [1]. In the extension study, patients were treated with 25 mg rituximab followed by 2 mL NaCl solution IT every sixth month for a total of five injections (Fig. 2). The dose was originally chosen from dose-ranging studies performed for treating CNS lymphoma, in which 25 mg as single injections intraventricularly was the highest dose without any tolerability issues [9].

#### Clinical assessments

Clinical assessments were performed to evaluate cognitive function (symbol digit modality test; SDMT), fatigue (fatigue scale for motor and cognitive function; FSMC), walking speed (6-min walk test and 25-foot walk test), and arm function (9-hole peg test). Adverse events (AEs) were recorded at each follow-up and as required.

### Statistical analysis

Statistical analyses were performed using IBM SPSS Statistics for Windows, version 23.0 (IBM Corp., Armonk, NY). Results are summarized with median, min. and max. values, together with interquartile range (IQR), and *p* values were calculated using Wilcoxon signed-rank test. Only patients completing the full 3-year period of both trials were included in the final data analysis. For patients that lost the ability to perform a walking test during the study due to disease progression, the value for walking speed obtained at the last measured point was used in the calculations.

### Data availability

The study protocol is available on request to the Principal Investigator (PI; AS). Raw data can be made available in a de-identified form upon written request to the PI under condition that an additional ethical approval is obtained from the Ethical Review Board.

## Results

### Adverse events

Table 2 summarizes all adverse events from both ITT-PMS trials. Mild to moderate vertigo and mild nausea was common in direct association with the injection procedure, commonly lasting 5–20 min and rarely needed symptomatic medication. Two cases of low-virulent bacterial meningitis caused by *Propionibacterium* were recorded, one in the ITT-PMS and one in the extension trial. Both were treated successfully with antibiotics. No other SAE were recorded.

**Table 2:** Adverse events recorded from the beginning of the 1-year ITT-PMS trial to the end of the 2-year extension trial for all participants

All events	
Any event	
Events—no. (no. per participant)	107 (4.7)
Patients with event—no. (%)	22 (96)
Severe adverse events	
Events—no. (no. per participant)	2 (0.09)
Patients with event—no. (%)	2 (9)
Bacterial meningitis—no. (%)	2 (9)
Death—no. (%)	0 (0)
Discontinuation because of adverse event—no. (%)	2 (9)
Moderate adverse events	
Events—no. (no. per participant)	23 (1.0)
Patients with event—no. (%)	15 (65)
*Vertigo, Upper respiratory infection, Urinary tract infection, Depression, Fall, Basalioma, Bladder stone, Deep venous thrombosis, Diabetes Mellitus type 2, Gastroenteritis, Vomiting*	
Mild adverse events	
Events—no. (no. per participant)	82 (3.6)
Patients with event—no. (%)	21 (91)
*Vertigo, Urinary tract infection, Paraesthesia, Diplopia, Upper respiratory infection, Rash, Headache, Nausea, Eczema, Fall, Fatigue, Fever, Fungal infection, Vomiting, Dry eye, Gastroenteritis, Hypertension, Labial herpes, Myalgia, Obstipation, Tremor*	
Frequency of adverse events	
Vertigo—no. (%)	23 (52)
Urinary tract infection—no. (%)	19 (43)
Upper respiratory infection—no. (%)	10 (26)
Paraesthesia—no. (%)	8 (13)
Nervous system disorders—Other, diplopia—no. (%)	6 (9)
Fall—no. (%)	4 (13)
Rash—no. (%)	4 (4)
Headache—no. (%)	3 (13)
Nausea—no. (%)	3 (9)
Vomiting—no. (%)	3 (13)
Depression—no. (%)	2 (4)
Eczema—no. (%)	2 (9)
Fatigue—no. (%)	2 (9)
Fever—no. (%)	2 (9)
Fungal infection—no. (%)	2 (4)
Gastroenteritis—no. (%)	2 (9)
Bacterial meningitis—no. (%)	2 (9)
Basalioma—no. (%)	1 (4)
Bladder stone—no. (%)	1 (4)
Deep venous thrombosis—no. (%)	1 (4)
Diabetes Mellitus type 2—no. (%)	1 (4)
Dry eye—no. (%)	1 (4)
Hypertension—no. (%)	1 (4)
Labial herpes—no. (%)	1 (4)
Myalgia—no. (%)	1 (4)
Obstipation—no. (%)	1 (4)
Tremor—no. (%)	1 (4)

### Clinical parameters

**Fig. 3:**
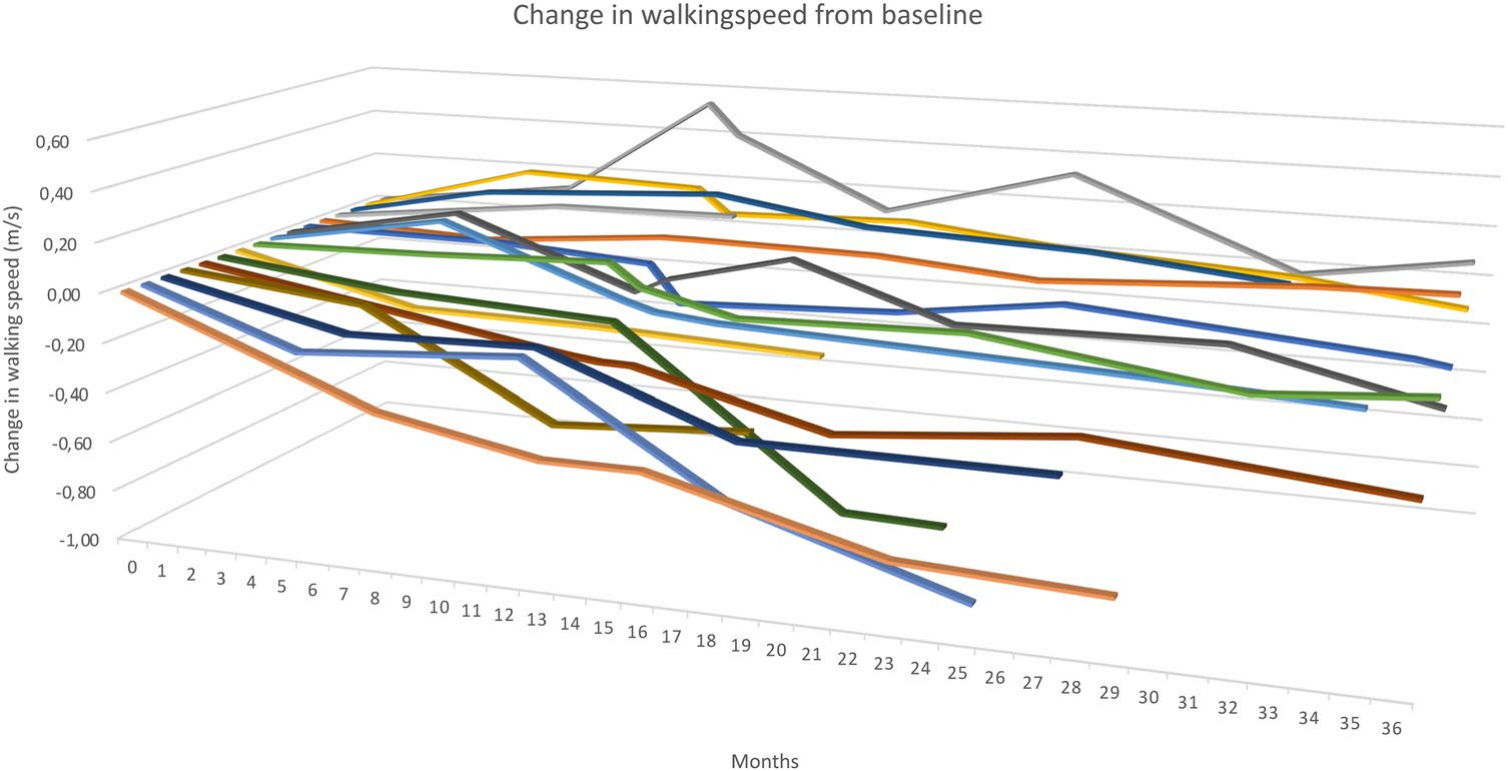
Development of walking speed in the patients, where this could be assessed at the onset of the ITT-PMS trial

The results of clinical assessments for the 15 participants that completed the full 3-year ITT-PMS trial are summarized in Table 3 and Fig. 3. Compared with baseline, walking speed deteriorated by 33% (*p* = 0.006) and SDMT improved by 1 point (*p* = 0.016). Other assessments did not change significantly over the trial period. As a sensitivity analysis, we also performed the same calculations on all patients entering the original ITT-PMS trial using the last measured value on each parameter even if the patient had discontinued during the course of the studies. We then obtained virtually identical median change of each parameter as reported in this study based on only completers of the two trials, not altering any significance level (data not shown).

**Table 3:** Clinical assessments

	*N*	Median	IQR	Max	Min	*Z* value	*p* value
Walking speed (m/s)							
Baseline ITT-PMS study	12	1.00	0.37	1.59	0.44		
Endpoint in extension trial	13	0.65	0.67	1.56	0.10		
Change	11	− 0.33	0.38	0.06	− 0.91	− 2.756	0.006
9HPT—Dominant hand							
Baseline ITT-PMS study	15	25.00	20.40	56.10	17.00		
Endpoint in extension trial	14	26.38	34.55	114.65	16.60		
Change	14	0.25	6.95	71.44	− 6.00	0.471	0.638
9HPT—non-dominant hand							
Baseline ITT-PMS study	15	27.40	12.45	58.50	21.35		
Endpoint in extension trial	15	30.30	18.55	56.90	19.45		
Change	15	2.30	8.04	30.50	17.65	0.568	0.570
FSMC							
Cognitive Score							
Baseline ITT-PMS study	15	32	12	42	12		
Endpoint in extension trial	15	32	14	48	20		
Change	15	1	11	13	− 12	0.220	0.826
Motor Score							
Baseline ITT-PMS study	15	41	7	50	19		
Endpoint in extension trial	15	36	10	48	29		
Change	15	− 4	8	12	− 15	− 1.592	0.111
Total Score							
Baseline ITT-PMS study	15	74	15	87	31		
Endpoint in extension trial	15	67	23	95	54		
Change	15	− 2	18	25	− 27	− 1.194	0.232
SDMT							
Baseline ITT-PMS study	15	48	15	65	22		
Endpoint in extension trial	15	49	19	68	23		
Change	15	1	6	13	− 6	2.404	0.016

Results from clinical assessments of the 15 participants that completed the full 3-year ITT-PMS + ITT-PMS extension studies

## Discussion

The original ITT-PMS study was designed to evaluate safety and feasibility of IT rituximab treatment with the idea to target compartmentalized inflammation in patients with purely progressive MS [1]. As exploratory endpoints, several clinical variables were assessed to detect trends of improvement or continued deterioration. Considering the insidious course of PMS, the addition of an extension trial with continued treatment was thought to increase the possibility to detect a clinically meaningful effect.

One important feature of PMS is the deterioration in walking ability. In the ITT-PMS trial walking speed was essentially unchanged but with the additional data from this 2-year extension trial, a statistically significant worsening could be demonstrated (Fig. 3). The magnitude in the degree of worsening (33%) is considered to be of clinical significance when evaluating walking tests [3]. To place our results in perspective, we compared the rate of deterioration with the data obtained in the phase 3 trial of interferon (IFN) beta-1a in progressive MS [2]. The rate of deterioration of walking speed in the IFN group (no raw data was available for the placebo group) over 2 years was 0.15 m/s corresponding to approximately 0.22 m/s over a 3-year period [3]. This is slightly lower rate than the 0.33 m/s deterioration observed in our study. Although a strict comparison is not possible, these data are in line with the conclusion that our data do not support a meaningful treatment effect of the IT rituximab treatment on walking speed.

Hand function has recently been emphasized as a better outcome measure for clinical trials in PMS, due to higher sensitivity in detection of positive treatment effects [4]. In the present study, the results of the 9-HPT were essentially unchanged, which could be interpreted as a successful outcome in a trial of PMS. However, the lack of a control group prohibits any strong conclusions about the effects on hand function. Fatigue, and to some extent cognitive functions, may on the other hand be more directly affected by meningeal inflammation. Unfortunately, there was no indication of a clinically relevant improvement in these parameters. A statistically significant improvement was seen in the SDMT, but the magnitude was not clinically meaningful, and a learning effect is expected.

The recently performed trials of ocrelizumab in primary progressive MS (the ORATORIO trial) imply that anti-CD20 therapy indeed may have a beneficial effect also in the progressive phase of MS [8]. In that respect, one has to consider that the study population at baseline in the ORATORIO trial was younger (mean age approximately 45 years), had a considerably lower EDSS (mean 4.7) and approximately 25% of the patients displayed Gadolinium-enhancing lesions at baseline. All these factors favor a possible treatment effect based on inflammatory activity, which all our patients lacked by definition. The small treatment effect seen on EDSS progression in the ORTORIO trial is, therefore, still not in complete disagreement with our data.

Intrathecally delivered rituximab treatment was generally well tolerated and no specific safety concern was observed related to rituximab per se. Two cases of low-virulent bacterial meningitis were recorded, likely the result of skin puncture and contamination from an area rich in sebaceous glands. Other side effects occurred immediately following the injection and were most likely due to an effect of altered dynamic of CSF pressure and flow in the ventricular system. Intraventricular administration was chosen for this study to secure adequate distribution in the full CSF compartment, but cannot be recommended for a possible clinical use because of its risk for infections. In case of any signs of positive effect from our study, we would have pursued further studies using the more conventional lumbar route for the delivery of the monoclonal antibodies.

The major shortcoming of this trial is the lack of control group. The invasive nature of the treatment precluded a larger trial directly and without clear indications of beneficial effects we chose to follow the initial study population for a longer period rather than initiating a controlled trial. Although we could document worsening in walking speed of most patients our data does not exclude a possible treatment effect in some individuals. With that in mind, the approach of IT administration of treatment in MS may still be an option to consider based on the idea of sequestered inflammation.
